# Higher Light Extraction Efficiency in Organic Light-Emitting Devices by Employing 2D Periodic Corrugation

**DOI:** 10.3389/fchem.2021.807867

**Published:** 2022-01-05

**Authors:** Yu Bai, Yahui Chuai, Yang Wang, Yingzhi Wang

**Affiliations:** Changchun University of Science and Technology, Changchun, China

**Keywords:** OLED (organic light-emitting diode), periodic (defect) structure, SPP (surface plasmon plaritons), TE mode and TM mode, light extraction enhancement

## Abstract

Photons trapped in the form of waveguide (WG) modes associated with the organic–organic interface and in the form of surface plasmon polariton (SPP) modes associated with the metallic electrode–organic interface result in a large energy loss in organic light-emitting devices (OLEDs). Introducing gratings onto the metallic electrode is especially crucial for recovering the power lost to the associated SPP modes. In our research, we demonstrate the efficient outcoupling of SPP modes in TE mode by two-dimensional (2D) grating, which cannot excited in one-dimensional (1D) grating OLED. This causes a 62.5% increase in efficiency from 2D grating OLED than 1D grating OLED. The efficient outcoupling of the WG and SPP modes is verified by the numerical simulation of both the emission spectra and the field distribution.

## 1 Introduction

In flat panel display and solid-state lighting, organic light-emitting devices (OLEDs) have great application potential. OLED has many advantages, such as energy efficiency, large viewing angle, low working voltage, fast response, and lightweight. Therefore, it is considered an ideal solution for future display and light sources. Although the internal quantum efficiency of OLED could nearly reach 100% now, its external quantum efficiency is still low, because of its low light extraction efficiency. Approximately 80% of the light emitted from OLED is absorbed by its internal waveguide (WG) mode, indium tin oxide (ITO) anode, and surface plasma polariton (SPP) generated between the organic layer/metallic cathode, and only 20% of the light gets to emerge out of OLED. In other words, OLED has a low light extraction efficiency. If we can solve the problem of light extraction efficiency of OLED, we can greatly enhance the external quantum efficiency of OLED (Gu et al., 1997; Erchak et al., 2001; Hobson et al., 2002; Kim et al., 2005).

One effective way of enhancing the light extraction efficiency of OLED is introducing periodic microstructures into the device. Periodic microstructures with period length between optical wavelength and subwavelength play a significant role in optical and optoelectronic devices (Moreland et al., 1982; Choi et al., 2005; Peng et al., 2005; Xie et al., 2008). Previously, we have reported that the method of laser two-beam interference can be used to directly ablate one-dimensional (1D) grating in the organic layer of OLED (Bai et al., 2011). By exciting the surface plasma effect and improving the WG effect of OLED, we can enhance its light extraction efficiency. However, for those devices that might produce surface plasma such as metallic cathodes of OLED, Worthing and Barnes (2001) pointed out that the production of surface plasma occurs in all directions of a plane, but 1D grating has directions, and only in half of the directions can surface plasma be coupled and excited; therefore, the coupling efficiency of surface plasma in 1D grating is low. Furthermore, for the WG mode of OLED, 1D grating can only reduce WG loss in specific directions and cannot help in other directions.

We introduced a two-dimensional (2D) grating structure into the organic layer of OLED in this research. Taking advantage of a higher coupling efficiency of SPP and WG mode in 2D grating structure, we obtained OLED with higher light extraction efficiency (Rigneault et al., 2000; Han et al., 2003; Giannattasio and Barnes, 2005; Jing et al., 2005; Wedge et al., 2005; Yang et al., 2005; Peng et al., 2006).

## 2 Experiment

In previous researches, we found that 350-nm periodic grating has SPP peak position of 607 nm. In order to leverage SPP to enhance light emitting, we need to choose optical materials with peak position of approximately 607 nm. DCJTB has a peak position of 610 nm (Xu et al., 2011); therefore, it was chosen.

A conducting polymer widely used in polymer LEDs as a hole-transport material, poly(N-vinyl carbazole) (PVK), is chosen, and it is spin-coated on the ITO glass for the fabrication of the corrugated hole transporting layer (HTL) in OLEDs. 4,4′,4″-Tris(3-methylphenylphe-nylamino)triphenylamine (m-MTDATA) was doped into the PVK with a concentration of 5% by weight to enhance the hole injection and transport of the HTL. The ablation experiments used a frequency-tripled Nd:YAG laser (Spectra-Physics Company) with 3-nm pulse width, 10-ns pulse length, 10-Hz repetition rate, and 355-nm wavelength. An ITO-coated glass substrate was cleaned with acetone and ethanol. The HTL was spin-coated at 4,000 rpm/s speed for 70-nm thicknesses. The sample was prebaked in vacuum for 30 min at 60°C to evaporate the organic solvent. Then, the sample was exposed by two beams, which were split from the UV laser with a beam size of 6 mm in diameter. The microstructure fabrication was conducted in air at room temperature using a single laser pulse for 1D grating. To obtain 2D grating, we prepared 1D grating first and rotated it by 60° and then did a second laser interference ablation. The periods for 1D and 2D grating are both 350 nm.

Prepared ITO substrates coated with corrugated HTL were immediately brought into a thermal evaporation chamber. Then, the 20-nm-thick HTL of N,N0 -diphenyl-N,N0-bis(1,10-biphenyl)-4,40-diamine was evaporated. After that, 4-(dicyanomethylene)-2-t-butyl-6-(1,1,7,7-tetramethyljulolidyl-9-enyl)-4H-pyran (DCJTB) with a concentration of 1% by weight was doped into 50-nm-thick emitting layer of tris-(8-hydroxyquinoline) aluminum (Alq3) for emission layer. At last, LiF (1 nm)/Al (100 nm) for cathode was evaporated. Here, all layers were prepared by thermal evaporation in a high vacuum system with the pressure of less than 5 × 10^−4^ Pa. The active area of the devices was 2 × 2 mm^2^. Their current density–voltage–luminance (J–V–L) characteristics were measured by Keithley 2400 programmable voltage–current source and Photo Research PR-655 spectrophotometer. The emission spectra at different observation angle were collected with a lens and then collimated and focused into the entrance slit of a 300-mm monochromator/spectrograph (SR-3031-A; Andor) to limit the angular acceptance to ∼1°. The spectrogram of the emission was recorded using a charge coupled device (iDus; Andor). The OLEDs were placed on a rotation stage with the grooves parallel to the rotation axis. All of the measurements were conducted in air at room temperature.

## 3 Discussion

**FIGURE 1:**
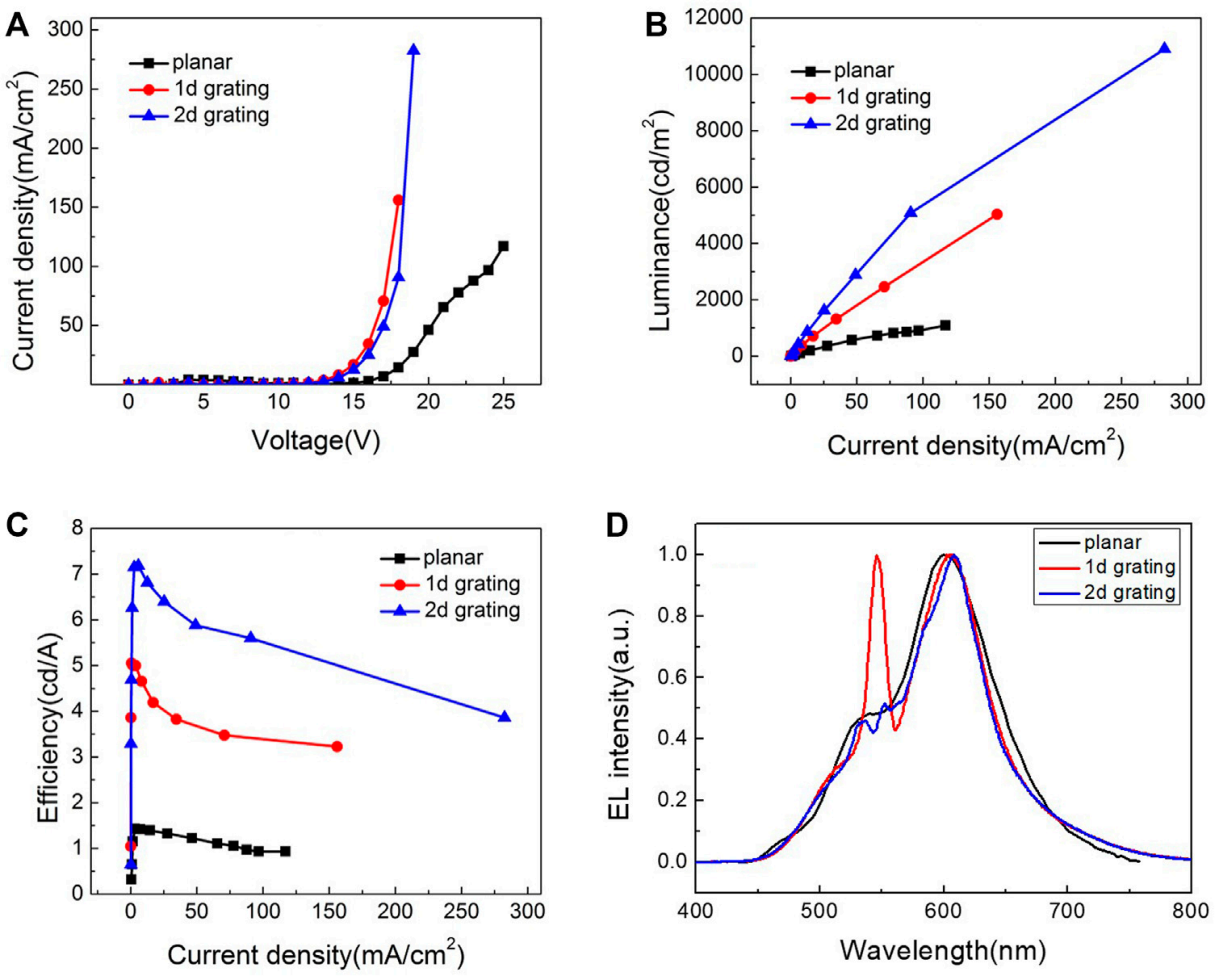
EL performance of the 1D grating, 2D grating, and planar OLEDs. Current density–voltage **(A)**, current density–luminance **(B)**, current density–efficiency **(C)** characteristics, and the EL spectra **(D)**.

Figure 1 shows the performance curve of the device and the EL spectra. From Figure 1A we can see, on both 1D and 2D devices, the current density increased significantly compared with the control flat panel device. That is because the grating microstructure increased the effective area of the device, and the laser ablation enhanced the transportation capacity of the hole-transport material. Figures 1B,C give the current density–luminance curve and current density–efficiency curve. We can see from them that both 1D and 2D grating devices have higher luminance and efficiency compared with flat panel devices. Between 2D and 1D grating devices, we can see the 2D device outperformed 1D device on current density, luminance, and efficiency. When the current density is 100 mA/cm^2^, the luminance of 2D device is 5,000 cd/m^2^, and the current efficiency is 5.53 cd/A, whereas the luminance of 1D device is 3,000 cd/m^2^, and current efficiency is 3.41 cd/A. When the device structure and other conditions are matched, 2D grating device has significantly better performance than 1D grating device, and this can only be explained by 2D grating having a higher coupling efficiency when it comes to surface plasma and WG mode.

To test our deduction, we simulated the spectra and optical field of 1D and 2D grating. We employed finite-difference time-domain method to simulate light transmission in the grating of OLED (Marcuse, 1974; Gedney, 1998). We used Drude model to represent the dielectric coefficient of metal and materials, and the refractive index of material was fitted into Drude model parameters from measurements of the ellipsometer. During simulation, for directions along the grating, we adopted periodic boundary condition, and for other boundaries, we adopted perfect matching layer to cut off. For the incident wave, we used modulated Gauss impulse whose central frequency is within the visible light band of our concern. We extracted the transmission light component and reflection light component at the end of computation and used the ratio of Poynting vector to represent transmission, reflection, and absorption.

**FIGURE 2:**
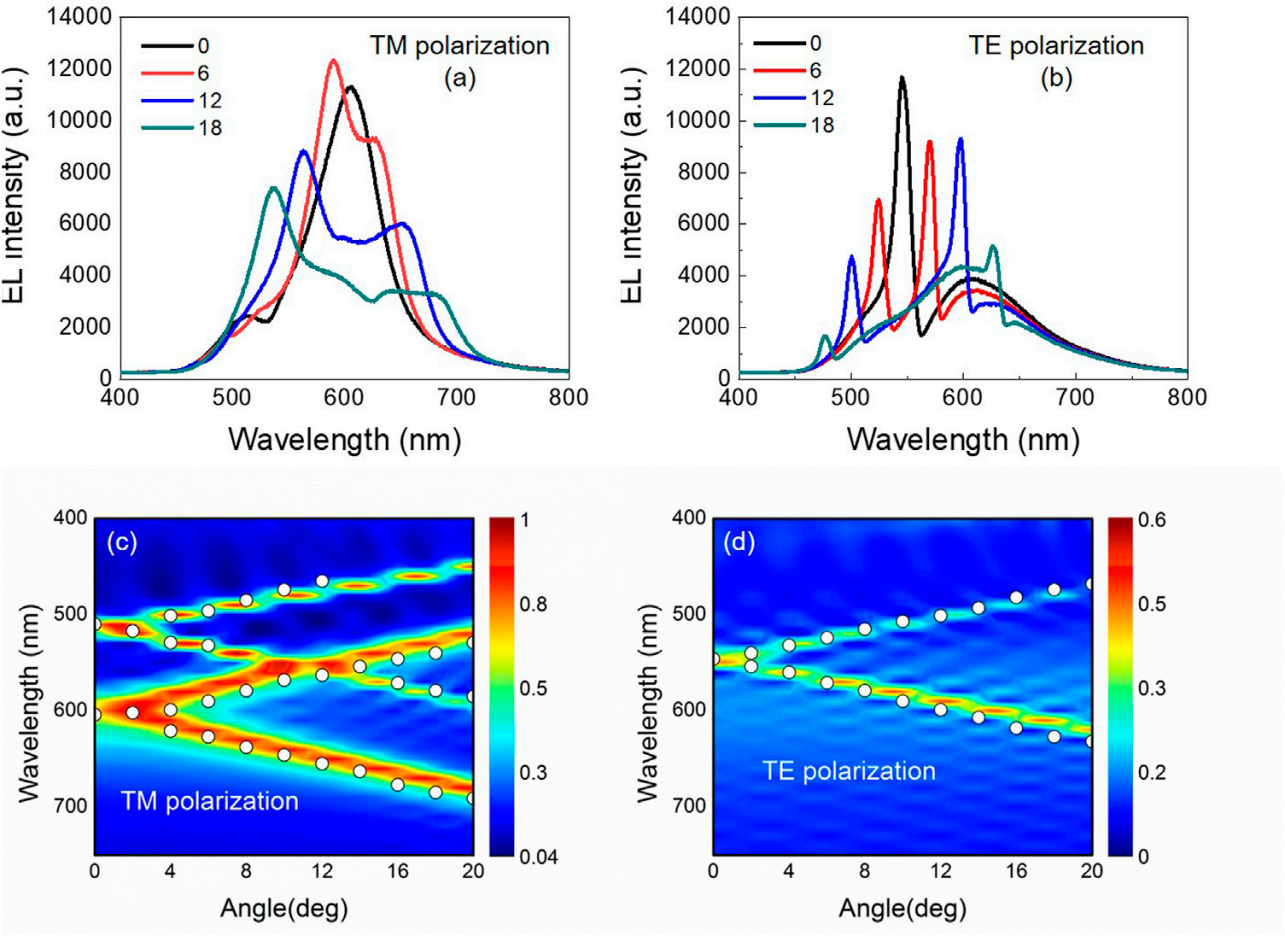
Measured EL spectra with TM **(A)** and TE **(B)** polarization at different observation angle from the corrugated OLEDs with 1D grating, and the wavelength versus incident angle for the calculated dispersion relation of the corrugated OLEDs for TM **(C)** and TE **(D)** polarization. The measured dispersion relation extracted from the EL spectra (circles) is also shown in **(C)** and **(D)**.

Figure 2 shows the angle-dependent spectrum and dispersion curve of 1D grating OLED in TM and TE mode, whereby white circles are dispersion curve obtained from actual measurement of angle-dependent spectrum. Putting actual data and simulated curve together, we can see that the two fit very well in terms of peak position and peak width at various angles in Figures 2C,D. Therefore, we consider the computation method for light transmission in grating OLED correct. In Figure 2, apart from the peak of DCJTB itself, in TM mode of Figure 2C, the device gives two additional peaks: 607 and 510 nm, and in TE mode of Figure 2D, the device gives one more peak: 550 nm. The additional 607-nm peak in TM mode is because the grating structure excited light in surface plasma mode inside the device, and the additional 510-nm peak is because the grating structure excited light in waveguided mode inside the device. In TE mode, there is only one additional peak; that is because the surface plasma mode cannot be excited in TE mode, and the single peak comes from grating structure exciting light in WG mode inside the device. Hence, for 1D grating, because of its directionality, it can only couple and excite surface plasma in half of the transmission directions, yielding a low coupling efficiency for surface plasma. Moreover, in WG mode of OLED, 1D grating can only reduce WG loss in specific directions, not in other directions.

**FIGURE 3:**
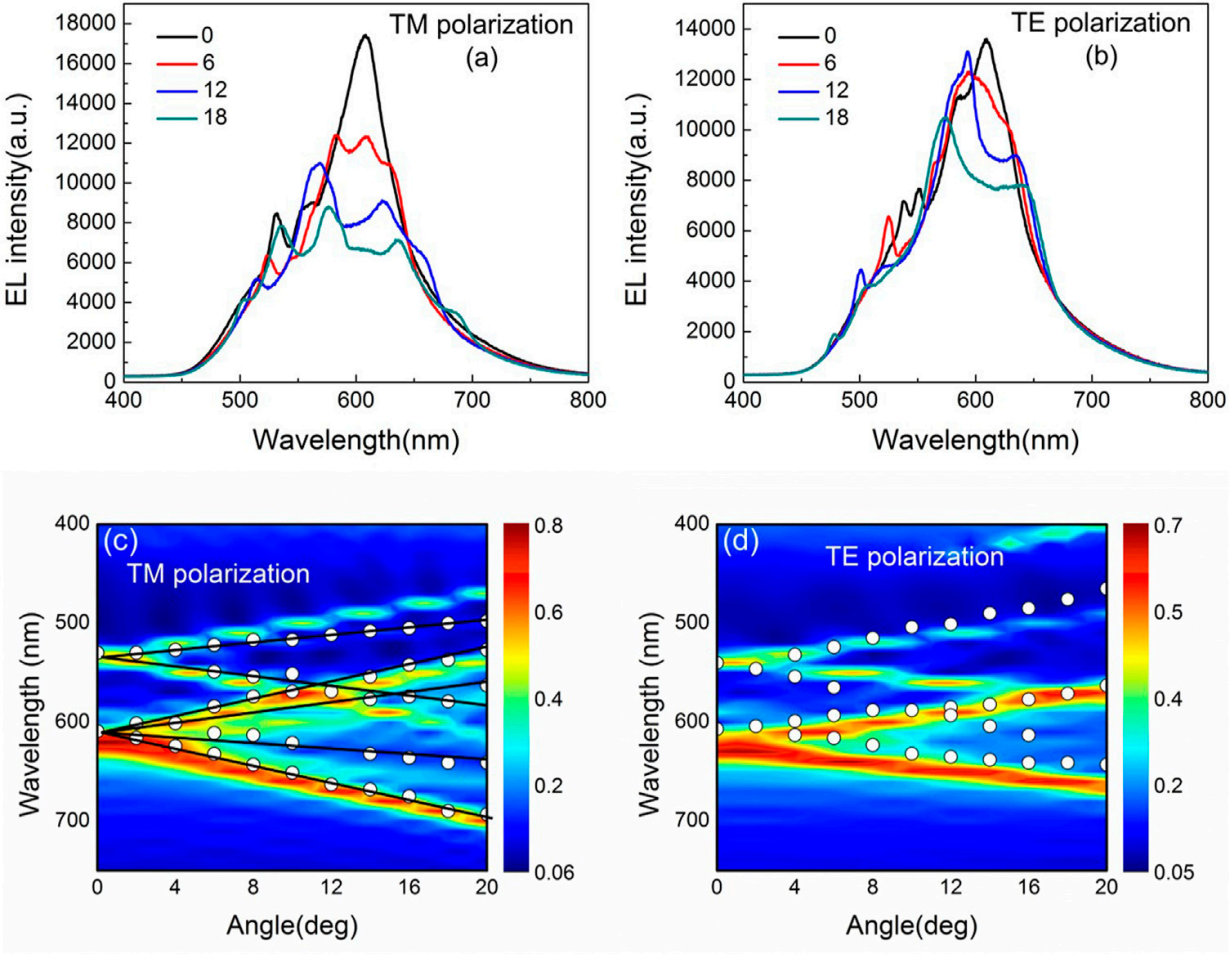
Measured EL spectra with TM **(A)** and TE **(B)** polarization at different observation angle from the corrugated OLEDs with 2D grating. And the wavelength versus incident angle for the calculated dispersion relation of the corrugated OLEDs for TM **(C)** and TE **(D)** polarization.

How about the coupling capacity for surface plasma in 2D grating? Figure 3 shows the angle-dependent spectrum curve and dispersion curve in TM and TE mode in 2D OLED. In Figures 3A,C at 0°, apart from the peak of DCJTB itself, there are two additional peaks at 538 and 630 nm, among which the 630-nm peak almost superposes on the 620-nm peak of DCJTB and is wider. When the observation angle increases, the two peaks split and shift with the increase in observation angle. For 1D OLED, when its additional peak splits as the observation angle increases, each peak split into two. But for the 2D device, the 630-nm peak does not split into two, but four. In Figures 3B,D at 0°, there are split peaks at 540 and 640 nm, respectively, among which the 640-nm peak is wider than the 540-nm peak.

**FIGURE 4:**
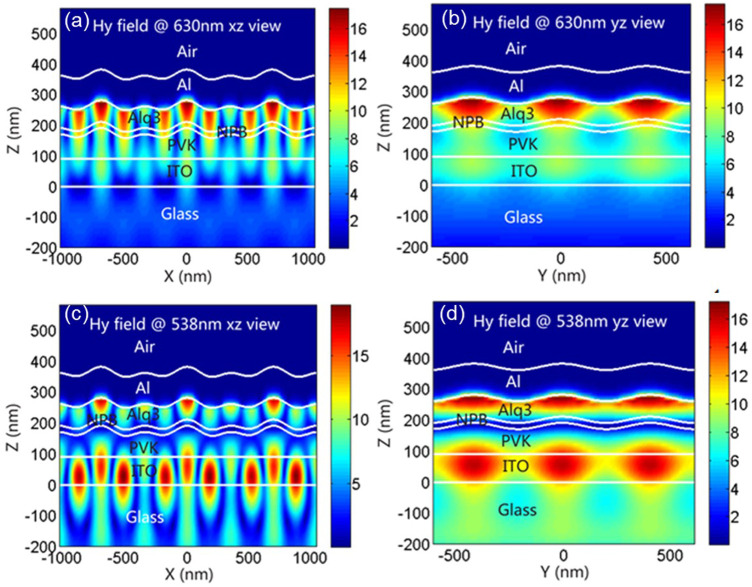
Distribution of the magnetic field intensity in the corrugated OLEDs at the wavelength of incident polarized light of 630-nm XZ view **(A)**, 630-nm YZ view **(B)**, 538-nm XZ view **(C)**, and 538-nm YZ view **(D)**, respectively.

To find out the reason for the formation of these additional peaks, we simulated and analyzed the field intensity in 2D OLED at observation angle of 0°. Figures 4A,B show the field intensity distribution of 630-nm peak at 0° in TM mode. We can see that, in both xz and yz directions, the greatest field intensity happens on the Al/Alq interface and in directions along the interface. That tells us the 630-nm peak is formed because the grating excites surface plasma (Worthing and Barnes, 2001; Liu and Tsai, 2002; Li and Ning, 2003; Gao et al., 2006; Van Oosten et al., 2010). And because the synthesis of *x* and *y* can be seen as any direction in the plane, we can see from the simulation of field intensity distribution that, in TM mode, 2D grating can couple and excite surface plasma in any direction. For the WG mode, as shown in Figures 4C,D in both xz and yz directions, the greatest field intensity happens in ITO and organic layers; therefore, the 538-nm peak is formed because the WG mode is excited. And as WG mode has been excited along *x* and *y* directions, it can be also considered that, in TM mode, 2D grating can couple and excite WG mode in any direction, yielding a better coupling efficiency.

**FIGURE 5:**
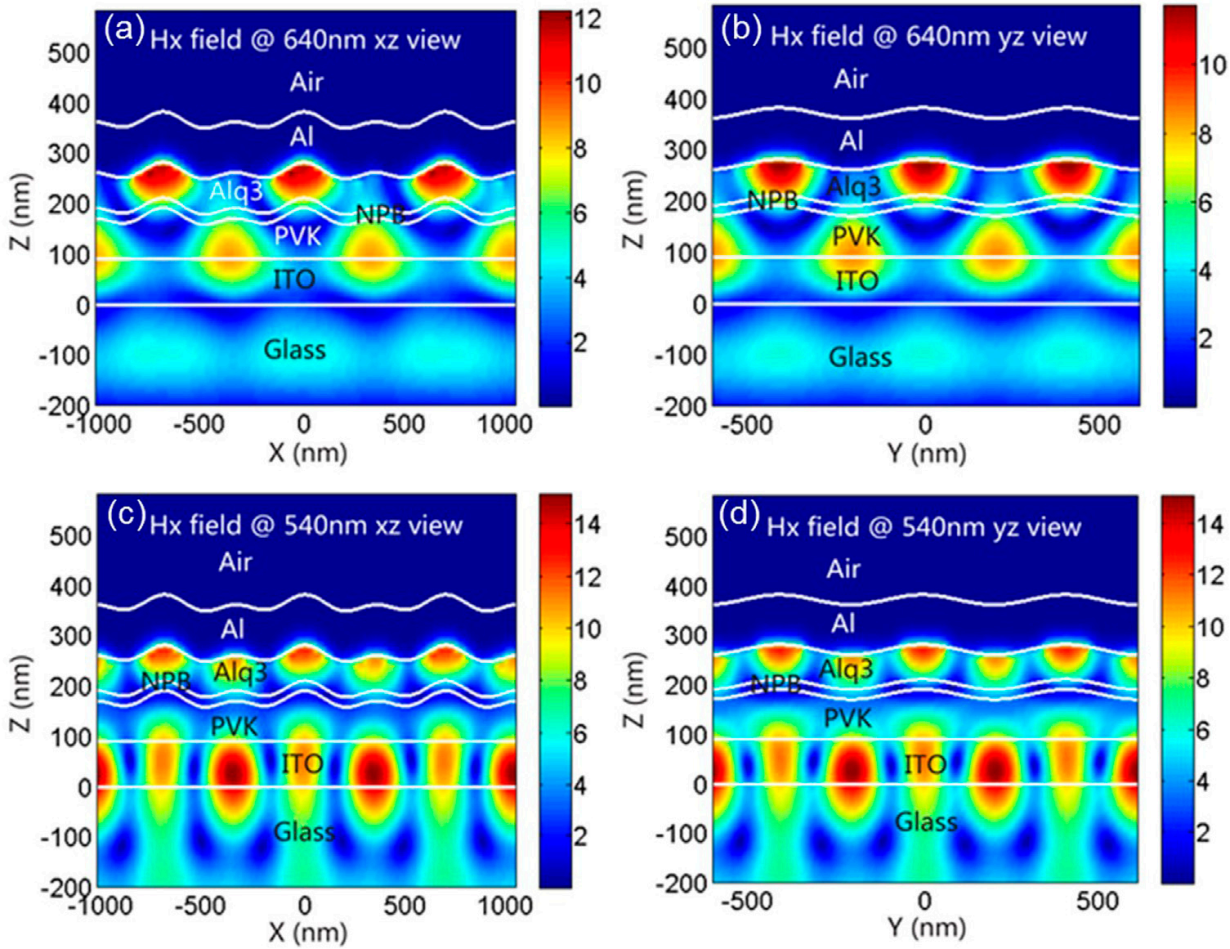
Distribution of the magnetic field intensity in the corrugated OLEDs at the wavelength of incident polarized light of 640-nm XZ view **(A)**, 640-nm YZ view **(B)**, 540-nm XZ view **(C)**, and 540-nm YZ view **(D)**, respectively.

Again, in TE mode, as shown in Figures 5A,B, in both xz and yz directions, although the field intensity is weak, the maximum intensity happens also at the Al/Alq interface and in directions along the interface, indicating that there are peaks of surface plasma in TE mode. For 1D grating OLED, surface plasma cannot be excited in TE mode; yet, we have observed surface plasma peaks in 2D grating OLED in TE mode.

We think that, for 2D grating in OLED, although one of the dimensions is in TE mode (hereby referred to as grating 1) and is incapable of exciting surface plasma mode, and the other dimension (hereby referred to as grating 2) because it intersects with grating a at 60°, we consider it, in our observation, as having two vectors: one is in parallel with grating a and the other is perpendicular to grating a. The vector parallel to grating a is in TE mode and therefore cannot excite surface plasma. But the vector perpendicular to grating a can be considered as being in TM mode and therefore can excite surface plasma. Therefore, in TE mode, weak surface plasma peaks were observed, which agrees with our actual measurement. Therefore, we think 2D grating OLED has a higher utilization efficiency for surface plasma than 1D grating OLED. In Figures 5C,D we can see, at peak 540 nm, the places with high field intensity concentrate in ITO and the organic layers; therefore, peak 540 nm is caused by WG mode excitation. Again, it can be excited along both *x* and *y* directions; therefore, it can also be seen as 2D grating can couple and excite WG mode in any direction, yielding a higher coupling efficiency.

## 4 Conclusion

We introduced 1D and 2D grating structures into the hole injection layer of OLED and, on that basis, obtained 1D and 2D grating OLED. Performance comparison shows that both 1D and 2D grating OLEDs yield better luminosity and efficiency than ordinary flat panel devices. At current density of 100 mA/cm^2^, the luminosity and efficiency of 1D grating OLED are 3.60 and 3.63 times those of the planar OLED, and the luminosity and efficiency of 2D grating OLED are 5.75 and 5.90 times those of the planar OLED. Because 2D grating has higher coupling and excitation efficiency for surface plasma and WG mode than 1D grating, 2D grating OLED brings greater enhancement on OLED luminosity and efficiency than 1D grating OLED; the luminosity and efficiency of 2D grating OLED increased by 59.7% and 62.5% than the 1D grating OLED.

## Data Availability

The original contributions presented in the study are included in the article/supplementary material, further inquiries can be directed to the corresponding author.
